# The dietary acid load is higher in subjects with prediabetes who are at greater risk of diabetes: a case–control study

**DOI:** 10.1186/s13098-019-0447-5

**Published:** 2019-07-01

**Authors:** Maryam Abshirini, Fariba Bagheri, Behzad Mahaki, Fereydoun Siassi, Fariba Koohdani, Maryam Safabakhsh, Gity Sotoudeh

**Affiliations:** 1 Department of Community Nutrition, School of Nutritional Sciences and Dietetics, Tehran University of Medical Sciences, Hojatdost Street, Naderi Street, KeshavarzBlv, Tehran, Iran; 2 Students’ Scientific Research Center, Tehran University of Medical Sciences, Tehran, Iran; 3 Department of Epidemiology and Biostatistics, School of Public Health, Kermanshah University of Medical Sciences, Kermanshah, Iran; 4 Department of Cellular and Molecular Nutrition, School of Nutritional Sciences and Dietetics, University of Medical Sciences, Tehran, Iran

**Keywords:** Dietary acid load score, Acid–base balance, Fasting blood glucose, Insulin resistance, Prediabetes

## Abstract

**Background:**

We aimed to examine the association of dietary acid load and prediabetes in a case–control study.

**Methods:**

This study recruited 297 subjects including 147 prediabetic subjects and 150 controls referred to diabetes screening center in Shahreza, Iran. Participants were frequency-matched by sex and age. Blood pressure, anthropometric measures and blood glucose levels were measured. Dietary intake was assessed using a validated 168-items food frequency questionnaire (FFQ). The dietary acid load scores including the potential renal acid load (PRAL) and net endogenous acid production (NEAP) were calculated based on nutrients intake. NEAP and PRAL scores were categorized by quartiles based on the distribution of controls. Logistic regression models were used to estimate multivariable odds ratio (OR) of prediabetes across the energy-adjusted and sex-specific quartiles of NEAP and PRAL scores.

**Results:**

Participants of control group in the higher quartiles of NEAP and PRAL tended to have higher body weight compared to the lower quartiles (P-trend < 0.04). After adjustment for multiple confounding variables, participants in the highest quartiles of NEAP and PRAL had increased OR for prediabetes (OR = 14.48 95% CI 5.64–37.19), and (OR = 25.61 95% CI 9.63–68.08) (P-trend < 0.001), respectively.

**Conclusion:**

Our data indicated subjects with prediabetes had diets with more acid-forming potential than control group. Our findings suggest that higher dietary acid load was associated with increased prediabetes morbidity. Interventional studies are warranted to elucidate the role of acidogenic diets in the development of prediabetes.

**Electronic supplementary material:**

The online version of this article (10.1186/s13098-019-0447-5) contains supplementary material, which is available to authorized users.

## Background

Prediabetes which is defined by glycemic profile higher than normal, but lowers than diabetes mellitus thresholds is a high-risk condition for the development of diabetes mellitus [1]. Prevalence of prediabetes is rapidly rising and it is predicted to affect over 470 million individuals by 2030 worldwide [2]. According to Iranian national survey of Tehran Lipid and Glucose Study, the rate of progression to prediabetes in the individual with normoglycaemia was 46 male and 38 female from every thousand male and female after 9 years follow-up [3]. People with prediabetes are at high risk of early forms of nephropathy, chronic kidney disease, diabetic retinopathy, and increased risk of macrovascular disease [4–6]. In subjects with prediabetes, lifestyle modification is considered the primary measure for diabetes prevention [7].

There is growing evidence that suggests the role of metabolic acidosis in the development of cardiometabolic abnormalities, particularly insulin resistance [8]. Furthermore, results from several previous cross-sectional studies have shown that hypocitraturia and more acidic urinary pH have been associated with risk of metabolic disorders such as insulin resistance and metabolic syndrome [9, 10]. In addition, low-grade metabolic acidosis has been linked to reduced insulin secretion [11]. Maintenance of pH balance mainly occurs as part of the body’s compensatory physiological responses [12], however, certain dietary factors due to their acid or base forming potentials have demonstrated to affect the acid–base status in the body [13, 14]. For example, western diets rich in animal proteins, which is known to be a major source for producing endogenous acid, is linked with increased metabolic acidosis [15]. In contrary, consumption of a dietary pattern high in fruits and vegetables has shown to promote the base-producing environment [16].

The acid-forming potential of diet is estimated by two methods: potential renal acid load (PRAL) [14], and net endogenous acid production (NEAP) [13] scores. These are established methods for predicting the acid loads from the composition of the consumed diet and are frequently used in epidemiologic studies. The dietary acid load provides a simple and useful tool for assessing the acidity of diet, as it correlates with urinary acid load measured from 24-h urine collection [13, 14].

The results from previous cohort studies stress the importance of acid-promoting diets in relevance to risk of type 2 diabetes mellitus (T2DM) [17, 18]. Nevertheless, the evidence on the association between the dietary acid load and insulin resistance and glucose hemostasis disturbance is still inconsistence and limited [19, 20]. The result of a cross-sectional study in healthy participants showed that PRAL and NEAP were positively associated with insulin resistance, whereas no association was observed with fasting glucose or glycated hemoglobin (HbA1c) [19]. In addition, these scores were not associated with insulin sensitivity and β-cell function or T2DM incidence in elderly men [20].

So far, the association between dietary acid load and prediabetes, a high-risk state for developing T2DM, has not yet been investigated. Therefore, the purpose of the present study was to examine the association between the dietary acid load score, assessed by both PRAL and NEAP and prediabetes morbidity in a case–control design. We hypothesized that participants in the case group will exhibit consuming more acid-producing diet than control group, resulting in a higher chance of prediabetes.

## Methods

Full details of the case–control study have been described in our previous study [21]. Briefly, this study was conducted from May to October 2014 at the diabetes screening center in Shahreza, Iran. Participants who were enrolled in the study were 300 men and women, above 30 years and at high risk of diabetes morbidity based on presenting at least one of the following conditions: overweight or obesity with body mass index (BMI) ≥ 25, family history of diabetes or existence of at least two symptoms of diabetes. Participants were placed into two groups: 150 participants with prediabetes as case and 150 participants as the control. Three participants in the case group were excluded from the study because they had energy intakes more than 3 standard deviations of the mean of energy intake, leaving 147 participants in the case group. The case and control groups were frequency matched by age and sex, and the age-frame for matching was 35–44, 45–54 and 55–65 years. The inclusion criteria for the prediabetic subjects were age above 30 years, FBG 100 to 125 mg/dl or 2-h OGTT of 140–199 mg/dl diagnosed no longer than 3 months before the interview. The inclusion criteria for the control group was age above 30 years, FBG < 100 mg/dl and 2-h OGTT of < 140 mg/dl during screening. Participants with the following criteria were excluded from the study: consuming alcohol, drug, and any tobacco products, having BMI ≥ 40 kg/m^2^, pregnant or lactating women and subjects with long-term dietary modification. Additionally, participants with the medical diagnosis of heart disease, diabetes, hypertension, dyslipidemia, renal or hepatic failure and multiple sclerosis were excluded from the study.

### Anthropometric, blood pressure and physical activity measurement

Height and weight were measured using stadiometer Seca 216 to the nearest 0.1 cm and Seca scale to the nearest 0.1 kg, respectively, while participants standing barefoot and wore undergarments. Waist circumference (WC) was measured using flexible tape at the midpoint between the lowest rib and the iliac crest. The BMI was calculated by dividing the weight in kilograms by the square of height in meter. Systolic and diastolic blood pressure was measured using a manual sphygmomanometer while sitting. Physical activity was assessed using the short form of the International Physical Activity Questionnaire (IPAQ) [22]. Physical activity was calculated by multiplying the metabolic equivalent (METs) measured in hours per week of each physical activity by the hours spent on the activity and summing of these values.

### Dietary assessment

To determine the usual dietary intake of the participants during the last year, 168 foods items food frequency questioner (FFQ) was used. The participants were asked to indicate the average daily, weekly or monthly frequency of consumption of each food items. The validity and reliability of this FFQ that used in Tehran Lipid and Glucose Study has been previously assessed on 132 apparently healthy adults in Tehran. The age and energy-adjusted correlation coefficients between the dietary intakes obtained from the FFQ and those from multiple 24-h dietary recalls were as the following: for protein 0.65 in men and 0.50 in women, for potassium 0.33 in men and 0.32 in women, for calcium 0.67 in men and 0.33 in women, for magnesium 0.65 in men and 0.39 in women, and for phosphorous 0.71 in men and 0.42 in women. These data revealed that the FFQ provides reasonable validity and reliability to assess the average long-term dietary intake of nutrients used for estimation of NEAP and PRAL [23]. Data obtained from FFQ were converted to grams in order to assess the nutrient and total energy intakes using the Nutritionist 4 software (First Databank Inc., Hearst Corp, San Bruno, CA) modified for Iranian foods.

### Laboratory assessment

Blood was collected after an overnight fasting (at least8 h). 2-h OGTT was performed in participants and the venous blood sample was drawn. Assessment of plasma glucose was performed at 546 nm wavelength using the photometric method (glucose oxidase method).

### Assessment of dietary acid load scores

The dietary acid load was assessed by previously established algorithms: NEAP [13] and PRAL [14]:

Net endogenous acid production (NEAP) (mEq/day) = (54.5 × protein [g/day]/potassium [mEq/day])_10.2.

Potential renal acid load (PRAL) (mEq/day) = (0.4888 × protein [g/day] +‏ 0.0366 × Phosphorus [mg/day]_0.0205 × Potassium [mg/day]_0.0125 × calcium [mg/day]_0.0263 × magnesium [mg/day].

To estimate the NEAP and PRAL, the nutrients intakes were adjusted for energy intake by residual method [24].

### Statistical analysis

Analyses were performed using SPSS, version 16.0 (SPSS Inc.). The Kolmogorov–Smirnov test was performed to check the normality of the data. NEAP and PRAL scores were categorized based on the distribution of control group. The characteristics of control group across the sex-specific and energy-adjusted quartiles of NEAP and PRAL were compared using the ANOVA for continuous variables and Chi square tests for categorical variables. Partial correlation was used to assess the existence of correlation adjusted by sex and energy intake between the NEAP and PRAL and food group intakes. Multivariate logistic regression was used to analyze the association of dietary acid load scores with prediabetes. The models were adjusted for age (years), sex (male or female), BMI (kg/m^2^), education (years), physical activity (MET/h/week), and energy intake (Kcal/day). These variables were chosen based on the difference that was observed between the two groups presented in Table 1 and previous studies [19, 20]. To test for trends, the median values of each quartile category of dietary acid load score were used as continuous variables. The results were considered significant if *P *< 0.05.

**Table 1:** Characteristics of control and pre-diabetic participants

Variables	Control	Pre-diabetic	P-value
	(n = 150)	(n = 147)	
Age (years)			
45<	57 (18.5)	55 (19.2)	0.9*
45–55	69 (23.9)	68 (22.9)	
55>	24 (8.1)	24 (8.1)	
Sex, n (%)			
Male	51 (17.2)	50 (16.8)	0.9*
Female	99 (33.3)	97 (32.7)	
Education (years)	6.9 ± 3.8	8.8 ± 4.8	< 0.001**
Weight (kg)	72.4 ± 11.4	77.7 ± 12.7	< 0.001*
BMI (kg/m^2^)	27.1 ± 3.6	29.3 ± 4.2	< 0.001*
WC (cm)	88.6 ± 9.9	94.1 ± 11.4	< 0.001*
PA level			
Low	37 (15.5)	89 (30)	< 0.001*
Medium	91 (30.6)	42 (14.1)	
High	22 (7.4)	16 (5.4)	
Energy intake (Kcal/day)	2278.4 ± 630.9	2499.2 ± 719.5	0.005^‡^
Fiber intake (g/day)	17.18 ± 5.16	13.62 ± 5.20	< 0.001**
Systolic blood pressure (mmHg)	118 + 10.9	122.6 + 12.9	0.002**
Diastolic blood pressure (mmHg)	73.4 + 7.7	78.6 + 10.5	< 0.001**
FBG (mg/dl)	82.1 ± 7.1	109.1 ± 6.5	< 0.001**
2-h OGTT (mg/dl)	120.4 ± 9.5	143.3 ± 18.1	< 0.001**
NEAP (mEq/day)	33.3 ± 10.8	45.5 ± 14.05	< 0.001**
PRAL (mEq/day)	− 17.3 ± 17.7	− 1.2 ± 21.6	< 0.001**

Data expressed as Mean ± standard deviation or number (%)

*BMI* body mass index, *WC* waist circumference, *PA* physical activity, *FBG* fasting blood glucose, *OGTT* oral glucose tolerance test, *NEAP* net endogenous acid production, *PRAL* potential renal acid load

* P-value is for Chi square test

** P-value is for Mann–Whitney test

^‡^Independent t test

### Results

Comparison the general characteristic of cases and controls are reported in Table 1; as it showed that participants with pre-diabetes had the higher mean of education, weight, WC, BMI, energy intake, blood pressure, FBG and 2-h OGTT compared with the control group (*P* value < 0.004). However, participants with pre-diabetes had less physical activity and fiber intake than control group (P-value < 0.001). The mean dietary acid load scores were significantly higher in participants with prediabetes compared with control group, NEAP (45.5 ± 14.05 vs. 33.3 ± 10.8 mEq/day, respectively, P-value < 0.001) and PRAL (− 1.2 ± 21.6 vs. − 17.3 ± 17.7 mEq/day, respectively, P-value < 0.001).

The characteristics of the control group across the quartiles of the NEAP and PRAL score based on the distribution of the control group are presented in Table 2. There were no significant differences in general characteristics across the quartiles of the NEAP. However, participants in the highest quartile of the NEAP tended to have higher weight and PRAL score than participants in the lowest quartile (P-trend < 0.04). Similarly, mean weight and NEAP score were significantly higher in the fourth quartile of PRAL relative to the first quartile (P-trend < 0.02).

**Table 2:** Characteristics of 150 control group across the sex-specific quartiles of energy-adjusted NEAP and PRAL score in a case–control study

Range	NEAP				
	Q1 (8.0–26.58)	Q2 (26.66–32.46)	Q3 (32.49–38.3)	Q4 (38.4–89.0)	P-trend
NEAP, median (mEq/day)	23.3	29.1	34.8	42.7	
No. of subjects	37	38	38	37	
Sex, n (%)^‡^					
Male	12 (8.4)	13 (8.8)	14 (8.4)	12 (8.4)	0.9
Female	25 (16.7)	25 (16.7)	24 (16.0)	25 (16.7)	
Age (years)^†^	47.5 ± 7.2	48.1 ± 8.3	46.9 ± 6.8	48.3 ± 6.6	0.8
Education (years)^†^	7.7 ± 4.0	6.5 ± 3.9	7.3 ± 3.5	6.3 ± 3.9	0.2
Dietary supplement use, n (%)^‡^					
Yes	5 (3.3)	3 (2.0)	9 (6.0)	3 (2.0)	0.1
No	32 (21.3)	35 (23.5)	29 (19.3)	34 (22.7)	
Weight (kg)^†^	67.4 ± 9.7	73.8 ± 9.3	76.0 ± 12.8	72.5 ± 12.0	0.03
Height (cm)^†^	160.4 ± 7.9	163.5 ± 8.4	165.3 ± 8.2	163.7 ± 8.8	0.057
WC (cm)^†^	85.9 ± 9.2	91.3 ± 10.1	88.9 ± 10.0	88.3 ± 9.7	0.4
BMI (kg/m^2^)^†^	26.2 ± 4.0	27.6 ± 2.9	27.7 ± 4.1	26.9 ± 3.1	0.4
PA (MET/h/week)^†^	1397.5 ± 1193.2	1462.4 ± 1311.1	1606.1 ± 1255.7	1788.5 ± 1105.9	0.1
Energy intake (Kcal/day)^†^	2257.1 ± 595.4	2373.7 ± 543.0	2302.0 ± 625.3	2177.4 ± 752.9	0.5
Systolic blood pressure (mmHg)^†^	119.1 ± 10.5	119.8 ± 10.1	117.6 ± 12.0	116.7 ± 10.9	0.2
Diastolic blood pressure (mmHg)^†^	72.5 ± 7.4	73.8 ± 8.1	74.6 ± 8.0	72.5 ± 7.6	0.8
FBG (mg/dl)^†^	82.8 ± 6.5	82.1 ± 6.9	82.1 ± 7.7	81.5 ± 7.5	0.4
2-h glucose (mg/dl)^†^	121.3 ± 9.0	120.9 ± 9.6	121.0 ± 9.7	118.2 ± 9.7	0.1
PRAL (mEq/day)^†^	− 37.4 ± 19.3	− 21.0 ± 5.1	− 11.4 ± 4.4	0.32.5 ± 9.8	< 0.001

Range	PRAL				
	Q1 (− 132.7, − 26.54)	Q2 (− 26.52, − 15.07)	Q3 (− 14.8, − 6.87)	Q4 (− 6.86, 32.9)	P-trend
PRAL, median (mEq/day)	− 32.8	− 21.6	− 11.8	− 2.6	
No. of subjects	38	38	37	37	
Sex, n (%)^‡^					
Male	11 (7.3)	14 (9.3)	11 (7.3)	15 (10.0)	0.6
Female	27 (18.0)	24 (16.0)	26 (17.3)	22 (14.7)	
Age (years)^†^	47.8 ± 7.3	47.3 ± 7.8	47.3 ± 7.1	48.4 ± 6.8	0.7
Education (years)^†^	7.1 ± 3.6	7.2 ± 4.1	7.1 ± 4.1	6.3 ± 3.6	0.3
Dietary supplement use, n (%)^‡^					
Yes	4 (2.7)	5 (3.3)	7 (4.7)	4 (2.7)	0.6
No	34 (22.7)	33 (22.0)	30 (20.0)	33 (22.0)	
Weight (kg)^†^	67.6 ± 9.1	74.3 ± 11.9	73.7 ± 11.4	74.2 ± 11.9	0.01
Height (cm)^†^	160.1 ± 7.7	164.9 ± 9.0	163.6 ± 8.2	164.5 ± 8.4	0.053
WC (cm)^†^	86.7 ± 9.2	89.7 ± 10.8	88.1 ± 9.1	90.1 ± 10.2	0.2
BMI (kg/m^2^)^†^	26.4 ± 3.8	27.2 ± 3.7	27.5 ± 3.8	27.3 ± 3.1	0.2
PA (MET/h/week)^†^	1600.7 ± 1244.9	1380.6 ± 1218.4	1473.0 ± 1228.4	1803.2 ± 1181.2	0.4
Energy intake (Kcal/day)^†^	2413.1 ± 633.0	2280.8 ± 584.4	2062.1 ± 580.4	2353.9 ± 688.1	0.3
Systolic blood pressure (mmHg)^†^	118.8 ± 10.5	119.6 ± 10.6	118.5 ± 11.6	116.4 ± 10.9	0.3
Diastolic blood pressure (mmHg)^†^	72.7 ± 7.5	73.6 ± 8.2	73.3 ± 7.6	73.7 ± 7.9	0.6
FBG (mg/dl)^†^	83.0 ± 6.6	80.8 ± 6.7	83.1 ± 7.1	81.5 ± 8.0	0.6
2-h glucose (mg/dl)^†^	122.8 ± 8.1	118.5 ± 9.5	123.3 ± 8.7	116.9 ± 10.3	0.057
NEAP (mEq/day)^†^	22.8 ± 4.0	29.8 ± 2.6	35.1 ± 3.3	45.9 ± 12.5	< 0.001

ANOVA and x^2^ tests were used for continuous and categorical variables, respectively

Energy-adjusted using the residual method

*NEAP* net endogenous acid production, *PRAL* potential renal acid load, *WC* waist circumference, *BMI* body mass index, *PA* physical activity, *MET* metabolic equivalent, *FBG* fasting blood glucose, *NEAP* net endogenous acid production

^†^Values are mean ± SD

^‡^Number of subjects having the characteristic with %

Furthermore, compared to those in the lowest quartile of PRAL score, participants in the highest quartile tended to have higher 2-h blood glucose, which was close to significant (P-trend = 0.057).

Multivariable-adjusted odds ratios (OR) for the association of energy-adjusted and sex-specific NEAP and PRAL with odds of prediabetes in the total study population is illustrated in Table 3. Higher NEAP was associated with an increased odds of prediabetes after adjustment for age and sex (OR = 15.22, 95% CI 6.24–37.0; P-trend = <0.001) (Model 1). Further adjustment for BMI (kg/m^2^), education (years), physical activity (MET/h/week), and energy intake (Kcal/day) did not change this association (OR = 14.48, 95% CI 5.64–37.19; P-trend < 0.001) (Model 2). Similarly, an increased OR for prediabetes was found across the quartiles of PRAL after adjustment for age and sex (Model 1), and was remained significant after adjustment for further potential confounding variables (OR = 25.61, 95% CI 9.63–68.08; P-trend < 0.001) (Model 2).

**Table 3:** Odds ratios (ORs) and 95% confidence intervals (CIs) of prediabetes according to sex-specific quartiles of energy-adjusted NEAP and PRAL score

Dietary acid load score	Q1	Q2	Q3	Q4	*P* for trend
No. of cases/control	Jul-38	Dec-37	25/38	103/37	
NEAP, median (mEq/day)	23.3	29.1	34.8	42.7	
Range	(8.0–26.72)	(26.66–32.49)	(32.28–38.31)	(38.41–89.0)	
OR (95% CI)					
Model 1	1.00 (Ref)	1.76 (0.62–4.97)	3.53 (1.36–9.15)	15.22 (6.24–37.0)	< 0.001
Model 2	1	1.71 (0.57–5.06)	2.27 (1.007–7.66)	14.48 (5.64–37.19)	< 0.001
No. of cases/control	22/78	36/40	31/18	58/14	
PRAL, median (mEq/day)	− 32.8	− 21.6	− 11.8	− 2.6	
Range	(− 132.7, − 26.54)	(− 26.52, − 15.07)	(− 14.8, − 6.87)	(− 6.86, 32.9)	
OR (95% CI)					
Model 1	1.00 (Ref)	3.65 (1.86–7.15)	9.24 (4.0–21.0)	29.83 (12.12–73.42)	< 0.001
Model 2	1	3.88 (1.89–7.98)	9.14 (3.75–22.29)	25.61 (9.63–68.08)	< 0.001

NEAP and PRAL were categorized into quartiles according to the distribution of the control group

NEAP for overall subjects (Q1: < 30.05, Q2: 30.05–37.68, Q3: 37.69–47.24 and Q4: ≥ 47.25); PRAL for overall subjects (Q1 < − 19.15, Q2: − 19.15 to − 8.19, Q3: − 8.18 to 2.56 and Q4: ≥ 2.57)

To test for a trend across quartiles, the median for each quartile category was used as a continuous variable

Model 1: adjusted for age and sex

Model 2: Model 1 further adjusted for BMI (kg/m^2^), education (years), physical activity (MET/h/week), and energy intake (Kcal/day)

*NEAP* net endogenous acid production, *PRAL* potential renal acid load

As expected, NEAP and PRAL scores were positively correlated with food group intakes including meat, processed meat, eggs and soft drinks intake (P-value < 0.05), while these scores were negatively correlated with intake of plant foods such as fruits, vegetables, and whole grains (P-value < 0.001). NEAP showed the inverse relationship with nuts and seeds intake (P-value = 0.006), however, this correlation was not observed with PRAL (Additional file 1: Table S1).

### Discussion

To best of our knowledge, this is the first study to examine the sex-specific relationship between NEAP and PRAL with the chance of prediabetes. The result of the present study demonstrated that NEAP and PRAL scores were higher among the subjects with prediabetes compared with controls, indicating a more acidogenic diet for them. In addition, NEAP and PRAL were positively associated with the odds of prediabetes independent of age, sex, BMI, physical activity, education, and energy intake.

Higher intake of foods with low acid-forming potential such as fruits and vegetables and replacing animal proteins intake with plant proteins appeared to be related to lower FBG, 2-h OGTT and HbA1c concentration [27, 28]. Our findings are in concordant with a study that reported the positive association between the dietary acid load and risk of T2DM [17, 18] and insulin resistance [19], which is mainly involved in the pathogenesis of multiple metabolic abnormalities, and known as a strong predictor of diabetes [36]. The E3N-EPIC cohort over 14 years in women [18] and pooled results of three prospective cohort studies including the Nurses’ Health Study (NHS), Nurses’ Health Study II (NHS2) and the Health Professionals’ Follow-up Study (HPFS) [17] reported a positive relationship between PRAL and NEAP with T2DM incidence. Accordingly, observational study among the healthy Japanese workers reported that PRAL and NEAP were positively associated with fasting insulin level and homeostatic model assessment of insulin resistance (HOMA-IR) score in participants with lower BMI (< 23 kg/m^2^), and NEAP score was related with the higher homeostatic model assessment of β-cell function (HOMA-β) score [19]. Although, results from the mentioned study [19] and study on female students [29] did not support a strong relationship between dietary acid load score and FBG and HbA1c level; these lack of associations may be partially explained by the fact that these studies conducted on apparently healthy and nondiabetic subjects who probably had sufficient β-cell function and insulin signaling that could maintain blood glucose concentration in optimal level. Further investigations are required to elucidate the potential role of dietary acid load on glucose tolerance in subjects with different glucose metabolism.

Furthermore, an observational survey with prospective follow-up of 18 years on Swedish non-diabetic elderly men revealed no association between the baseline FBG and β-cell function with dietary acid load. It was probably the similarity between age, gender, and ethnicity of participants that led to less variation in dietary acid load scores and caused non-significant results [20].

In some of previous studies the strength of the associations between the diet-dependent acid load and risk of T2DM were inconsistent across different indices of dietary acid load, and sex-difference was suggested as potential confounder in these associations. A recent meta-analysis of seven cohort prospective observational studies suggested a linear and positive association between NEAP and animal protein-to-potassium ratio (A:P) and risk of T2DM, however, the association between PRAL and T2DM incidence was U-shaped and nonsignificant [26]. Data from The Japan Public Health Center based Prospective Study on men and women aged 45–75 years showed that PRAL, but not NEAP, was related with the risk of T2DM; and association was confined only to younger men [25]. Meanwhile, subgroup analysis in three US [17] and seven cohort studies [26] showed that association between dietary acid load score and T2DM were only evident among the women. Given that observed associations between PRAL and NEAP with prediabetes in current study were sex-specific, thus; acid-promoting diets may be associated with increased chance of prediabetes in both genders in a similar manner. Future studies are required to determine the potential sex difference in the development of prediabetes induced by diet-dependent acid load.

There are some mechanisms of action that have been suggested to link the dietary acid load to prediabetes. One of the possible mechanisms responsible for the association between high-dietary acid load and the risk of prediabetes is increased production of acid-forming metabolites, which can lead to the release of plasma glucocorticoid, which consequently results in impairment of insulin sensitivity [30, 31]. Moreover, nutrients such as potassium and magnesium, mostly derived from plant-based foods have the major role in acid–base equilibrium [32]. In this regard, diets with the shortage of fruits and vegetables and deficient in these nutrients have shown to drive the pH-balance towards acidosis, that impairs the β-cell response and lead to insulin resistance [11]. Finally, the high acid load may also induce significant degrees of insulin dysfunction due to increased urinary secretion of minerals that are essential in insulin function such as calcium and magnesium [33–35].

Our study had several strengths and limitations. First of all, the present study was the first study to examine the association between the dietary acid load and the chance of prediabetes in a case–control design. Additionally, including the wide range of data on confounding variables and conducting the sex-specific analysis of dietary acid load of the study participants helped to reach an independent association. However, because the dietary intake was assessed by FFQ, the recall bias and measurement error were inevitable. Another particular concern is that we did not control for kidney function of participants which is critical in determining the acid–base hemostasis. Therefore, further studies that extensively include urinary markers of acid–base status and kidney function are warranted to clearly illustrate the observed associations. Pre-diabetic participants were asked to report their dietary intake of the year before the diagnosis of pre-diabetes. However, they were aware of their condition which might affect dietary responses. Moreover, the present study included participants who are at high risk of diabetes, thus interpretation of our findings could not be applicable to general population without any risk. Finally, given the case–control design of the study, we were unable to conclude the causal relationship, whether the higher dietary acid load leads to prediabetes development or vice versa. Hence, the interventional investigations are required to establish the role of diet acidity as a cause of prediabetes.

## Conclusion

In conclusion, the present study showed a positive and independent association between the dietary acid load scores and chance of prediabetes. Thus, targeting an improvement in dietary acid–base balance might be a useful strategy for prediabetes prevention. However, further studies, particularly with prospective design are recommended to confirm our findings.

## Additional file

**Additional file 1: Table S1.** Food intake and their correlations with dietary acid load scores.

## Data Availability

Not applicable.

## Abbreviations

FFQ: food frequency questionnaire
PRAL: potential renal acid load
NEAP: net endogenous acid production
OR: odds ratio
BMI: body mass index
FBG: fasting blood glucose
T2DM: type 2 diabetes mellitus
HbA1c: glycated hemoglobin
OGTT: 2-h oral glucose tolerance test
WC: Waist circumference
IPAQ: International Physical Activity Questionnaire
HOMA-β: homeostatic model assessment of β-cell function
HOMA-IR: homeostatic model assessment of insulin resistance
NHS: Nurses’ Health Study
HPFS: Health Professionals’ Follow-up Study
AHEI: Alternative Healthy Eating Index
A:P: animal protein-to-potassium ratio
