# Supplementary material for: The dietary acid load is higher in subjects with prediabetes who are at greater risk of diabetes: a case–control study
Source: Diabetol Metab Syndr. 2019 Jul 1;11:52. doi: 10.1186/s13098-019-0447-5 (PMC6604202; doi:10.1186/s13098-019-0447-5)
Supplement: Supplementary file 1 — Additional file 1: Table S1. Food intake and their correlations with dietary acid load scores. [file 13098_2019_447_MOESM1_ESM.docx]

| Food intake and their correlations† with dietary acid load scores. | | | | | | | | | |
| --- | --- | --- | --- | --- | --- | --- | --- | --- | --- |
| **PRAL** | | **NEAP** | | | | Mean± SD  Median (IQR) | | | **Food intake** |
| *P* | *r* | | *P* | *r* | |  | |  | |
| 0.001> | 0.49 | | 0.001> | | 0.57 | | 71.64(61.46) | Meat (g/d)†† | |
| 0.001> | 0.31 | | 0.001> | | 0.32 | | 4.60(13.77) | Processed meat(g/d) †† | |
| 0.8 | -0.01 | | 0.8 | | -0.01 | | 9.48(16.75) | Fish(g/d) †† | |
| 0.2 | 0.06 | | 0.7 | | 0.02- | | 477.8(292.20) | Dairy products(g/d) †† | |
| 0.001> | 0.28 | | 0.001> | | 0.24 | | 30.00(36.21) | Eggs(g/d) †† | |
| 0.006 | 0.15- | | 0.001> | | 0.23- | | 6.00(24.08) | Whole grains(g/d) | |
| 0.001> | -0.41 | | 0.001> | | -0.47 | | 448.32±289.55 | Vegetables(g/d)* | |
| 0.001> | -0.34 | | 0.001> | | -0.42 | | 249.35(185.98) | Fruits(g/d) †† | |
| 0.1 | -0.09 | | 0.006 | | -0.15 | | 7.86(10.40) | Nuts and seeds(g/d) †† | |
| 0.004 | 0.14 | | 0.04 | | 0.11 | | 9.33(35.00) | Soft drinks(g/d) †† | |
| 0.3 | -0.05 | | 0.1 | | -0.07 | | 480.0(486.50) | Coffee and tea(g/d) †† | |

SD: standard deviation; IQR: interquartile range; NEAP: net endogenous acid production; PRAL: potential renal acid load

Mean± SD for variables with normal distribution *

†† Median (IQR) for variables with non-normal distribution

†Partial correlation was adjusted for sex and energy intake.
